# Exogenous nitric oxide stimulates the odontogenic differentiation of rat dental pulp stem cells

**DOI:** 10.1038/s41598-018-21183-6

**Published:** 2018-02-21

**Authors:** Soichiro Sonoda, Yu-feng Mei, Ikiru Atsuta, Atsushi Danjo, Haruyoshi Yamaza, Shion Hama, Kento Nishida, Ronghao Tang, Yukari Kyumoto-Nakamura, Norihisa Uehara, Toshio Kukita, Fusanori Nishimura, Takayoshi Yamaza

**Affiliations:** 1 Department of Molecular Cell Biology and Oral Anatomy, Division of Oral Sciences, Kyushu University Graduate School of Dental Science, Fukuoka, Japan; 2 Section of Periodontology, Division of Oral Rehabilitation, Faculty of Dental Science, Kyushu University, Fukuoka, Japan; 3 Research Fellow of Japan Society for the Promotion of Science, Tokyo, Japan; 4 Key Laboratory of Oral Diseases of Jiangsu Province and Stomatological School of Nanjing Medical University, Nanjing, Jiangsu China; 5 Department of Pediatric and Preventive Dentistry, The Affiliated Stomatological Hospital of Nanjing Medical University, Nanjing, Jiangsu China; 6 Section of Implant and Rehabilitative Dentistry, Division of Oral Rehabilitation, Faculty of Dental Science, Kyushu University, Fukuoka, Japan; 7 Department of Oral and Maxillofacial Surgery, Faculty of Medicine, Saga University, Saga, Japan; 8 Department of Pediatric Dentistry, Division of Oral Health, Growth & Development, Kyushu University Graduate School of Dental Science, Fukuoka, Japan; 9 Kyushu University School of Dentistry, Fukuoka, Japan

## Abstract

Nitric oxide (NO) is thought to play a pivotal regulatory role in dental pulp tissues under both physiological and pathological conditions. However, little is known about the NO functions in dental pulp stem cells (DPSCs). We examined the direct actions of a spontaneous NO gas-releasing donor, NOC-18, on the odontogenic capacity of rat DPSCs (rDPSCs). In the presence of NOC-18, rDPSCs were transformed into odontoblast-like cells with long cytoplasmic processes and a polarized nucleus. NOC-18 treatment increased alkaline phosphatase activity and enhanced dentin-like mineralized tissue formation and the expression levels of several odontoblast-specific genes, such as runt related factor 2, dentin matrix protein 1 and dentin sialophosphoprotein, in rDPSCs. In contrast, carboxy-PTIO, a NO scavenger, completely suppressed the odontogenic capacity of rDPSCs. This NO-promoted odontogenic differentiation was activated by tumor necrosis factor-NF-κB axis in rDPSCs. Further *in vivo* study demonstrated that NOC-18-application in a tooth cavity accelerated tertiary dentin formation, which was associated with early nitrotyrosine expression in the dental pulp tissues beneath the cavity. Taken together, the present findings indicate that exogenous NO directly induces the odontogenic capacity of rDPSCs, suggesting that NO donors might offer a novel host DPSC-targeting alternative to current pulp capping agents in endodontics.

## Introduction

Primary odontoblasts, which are cranial neural crest cell-derived ectodermal mesenchymal stem cells present in the dental papilla, form the primary dentin during tooth development and secondary dentin after tooth eruption. When the dentin detects various noxious stimuli, such as bacterial toxins, mechanical trauma, and/or tooth preparation, tertiary dentin is formed at the dentin-pulp border beneath the injured dentin as part of the tissue repair process^1^. Tertiary dentinogenesis is approved for use after vital pulp therapy. Tertiary dentin is divided into reactionary and reparative dentin according to the response of the primary odontoblasts. Reactionary dentin is formed by the post-mitotic primary odontoblasts that survive after tooth injury, while reparative dentin is reconstructed by newly differentiated odontoblasts, which are recruited from odontogenic stem/progenitor cells.

A novel odontogenic mesenchymal stem cell (MSC) population, the dental pulp stem cells (DPSCs), have been successfully isolated from the dental pulp tissue of permanent teeth^2^. DPSCs are a clonogenic population that exhibits stem cell-like properties, including self-renewal capacity, high cell proliferation ability, and multi-differentiation capacity^3^. DPSCs express runt related factor 2 (*RUNX2*) and dentin sialophosphoprotein (DSPP) genes under odontogenic culture conditions, and they exhibit a capacity for forming dentin-pulp complex in a subcutaneous transplantation system using hydroxyapatite and tricalcium phosphate as a carrier^2^. DPSCs are located in the perivascular niche around the capillaries in dental pulp tissues^4^. Therefore, DPSCs recruited from the perivascular niche may be involved in the reconstruction of reparative dentin.

Nitric oxide (NO) is biosynthesized from L-arginine through reactions catalyzed by NO synthases (NOSs), including endothelial NOS, neuronal NOS, and inducible NOS (iNOS), and NO acts as a biological regulator under both physiological and pathological conditions^5^. Recently, synthetic NO-releasing compounds, so called NO donors, were developed, and have been applied in a variety of biological and medical fields^6^. Generation of exogenous NO using S-nitroso-N-acetyl-penicillamine (SNP) as a NO donor directly accelerates the differentiation of cultured mouse calvaria-derived osteoblasts^7^ and embryonic stem cells^8^. NO donors, or nitric oxide-releasing compounds (NOCs), are NO-containing zwitterions that can generate NO continuously^9^. In contrast, synthetic NO scavengers, such as 2-phenyl-4,4,5,5-tetramethylimidazoline-1-oxyl-3-oxide (PTIOs), can neutralize NO directly and completely via direct radical-radical reactions with NO^10,11^, and are an essential tool for analyzing the biological actions of NO.

It is not known whether NO directly affects the differentiation of odontoblast stem/progenitor cells *in vitro* and *in vivo*. Therefore, to determine the direct effects of NO on the differentiation and function of odontoblast stem/progenitor cells, we isolated DPSCs from neonatal rat incisors (rDPSCs), and then examined the effects of the NO donor 1-hydroxy-2-oxo-3,3-bis(2-aminoethyl)-1-triazen (NOC-18) and the NO scavenger 2-(4-carboxyphenyl)-4,4,5,5-tetramethylimidazoline-1-oxyl-3-oxide sodium salt (carboxy-PTIO) on the viability and odontogenic capacity of rDPSCs by *in vitro* cytodifferentiation analyses. Further mechanistic studies were demonstrated by the gene expression assay of *Rela* (*NF-κB p65*) and NF-κB inhibitor test using pyrrolidine dithocarbamate (PDTC). Furthermore, we examined whether NOC-18 accelerated the dentin-pulp repair process, tertiary dentinogenesis, *in vivo* using a tooth preparation model in rats, by histological analyses.

## Results

### Isolation and characterization of rDPSCs

**Figure 1:**
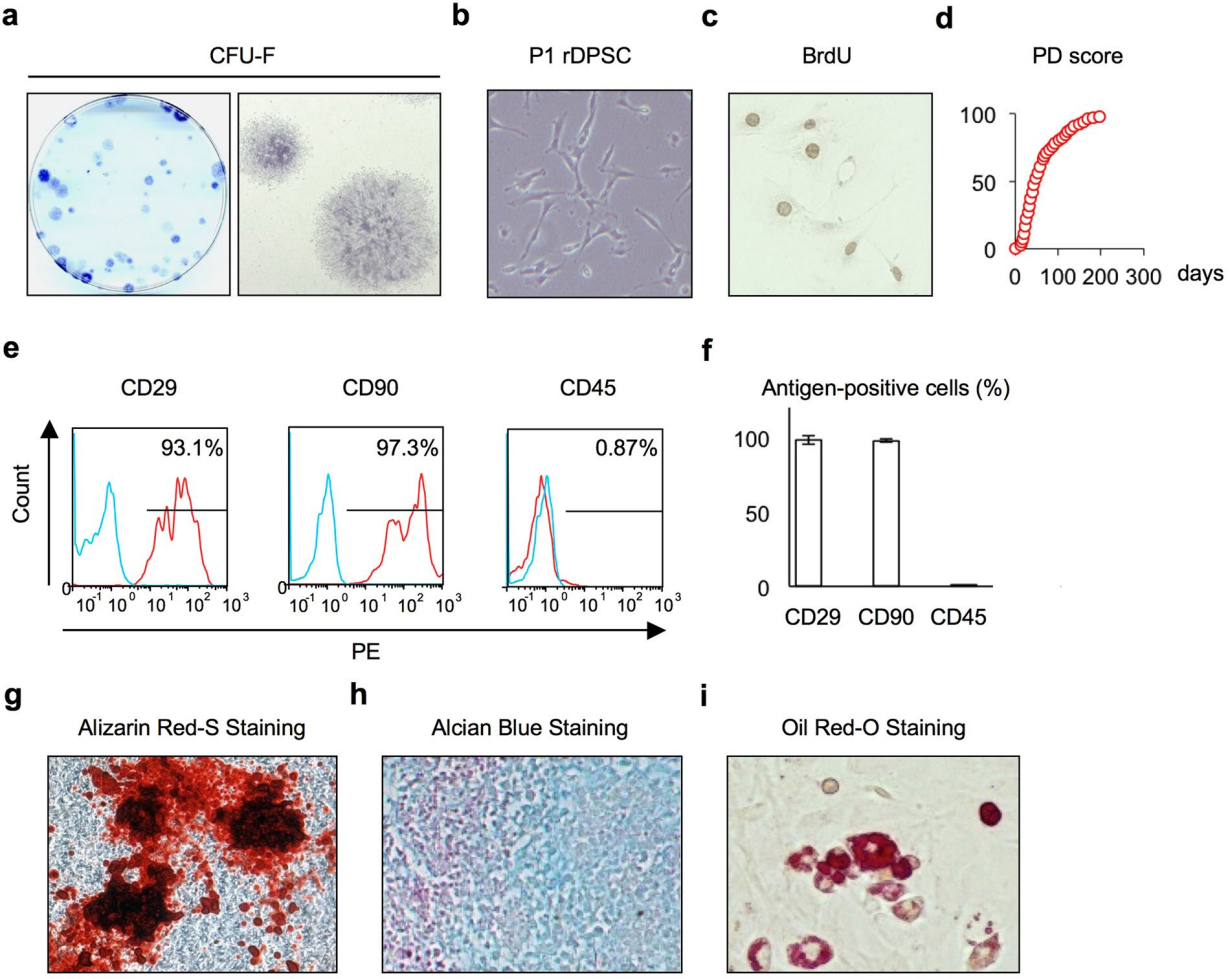
Characterization of rat dental pulp stem cells (rDPSCs). (**a**) Colony-forming capability of rDPSCs as shown by toluidine blue staining. Representative images of colony-froming unit fibroblats (CFU-Fs) in a culture dish (left panel) and fibroblastic colonies (right panel). (**b**) Representative image of passage 1 (P1) rDPSCs. (**c**) Representative image of rDPSCs with BrdU-positive nuclei. (**d**) Population doubling (PD) score of rDPSCs. (**e,f**) Immunophenotype assay by flow cytometric analysis. Red histograms: cell surface antigen-specific antibodies; blue histograms: subclass-matched control antibodies. Percentiles indicate the average for each antigen. PE: phycoerythrin (**e**). Percentiles of cell surface antigen-positive cells among total cells (n = 3 per group). Graph bars are the means ± standard error of the mean (SEM) (**f**). (**g–i**) Multipotency of rDPSCs. Odontogenic/osteogenic (**g**), chondrogenic (**h**), and adipogenic (**i**) capacity.

The cells isolated from the dental pulp tissues of rat incisors were capable of forming adherent clonogenic colony clusters of different sizes and densities (Fig. 1a). These clusters consisted of spindle-shaped cells (Fig. 1a). Passage 1 (P1) rDPSCs (Fig. 1b) showed high cell proliferative capacity by BrdU incorporation and population doubling assays (Fig. 1c,d). Flow cytometric analysis showed that the P1 cells were positive for the MSC surface markers CD29 and CD90 and negative for the hematological marker CD45 (Fig. 1e,f). A multipotent assay showed mineralized nodule formation by Alizarin red-S staining (Fig. 1g), proteoglycan accumulation by Alcian blue staining (Fig. 1h), and lipid accumulation by Oil-red-O staining (Fig. 1i) in rDPSCs under specific culture conditions. These findings indicated that our isolated cells were rDPSCs according to the minimal criteria for MSCs^12^.

### The NO scavenger carboxy-PTIO reduces the viability of rDPSCs, whereas the NO donor NOC-18 does not

To examine effects of exogenous NO on the viability of DPSCs, these cells were stimulated with the NO donor NOC-18 (0, 0.1, 1.0, and 10 μM), and cell viability was measured by the WST assay at 1, 2, and 3 days after stimulation. The viability of NOC-18-treated rDPSCs was similar to that of control rDPSCs without NOC-18 treatment (Supplementary Figure 1a). Conversely, the viability of rDPSCs treated with NOC-18 (10 μM) in the presence of the NO scavenger carboxy-PTIO (100 μM) was significantly lower than that of the control rDPSCs on day 1 and 2 after stimulation (Supplementary Figure 1b). These findings suggested that endogenous NO, but not exogenous NO, was involved in maintaining the viability of rDPSCs.

### NOC-18 induces odontoblastic features in rDPSCs

To examine the effects of exogenous NO on the morphology of DPSCs, these cells were incubated with or without 10 μM NOC-18 for 3 days, and the cell membrane was stained. While untreated rDPSCs appeared as spindle-shaped fibroblastic cells, some of the NOC-18-treated rDPSCs showed odontoblast-like features, with ovoid-shaped cell bodies, long cytoplasmic processes, and a polarized nucleus (Supplementary Figure 1c). These findings suggested that exogenous NO may commit undifferentiated DPSCs to odontoblast-lineage cells.

### NOC-18 enhances the odontoblast differentiation of rDPSCs

**Figure 2:**
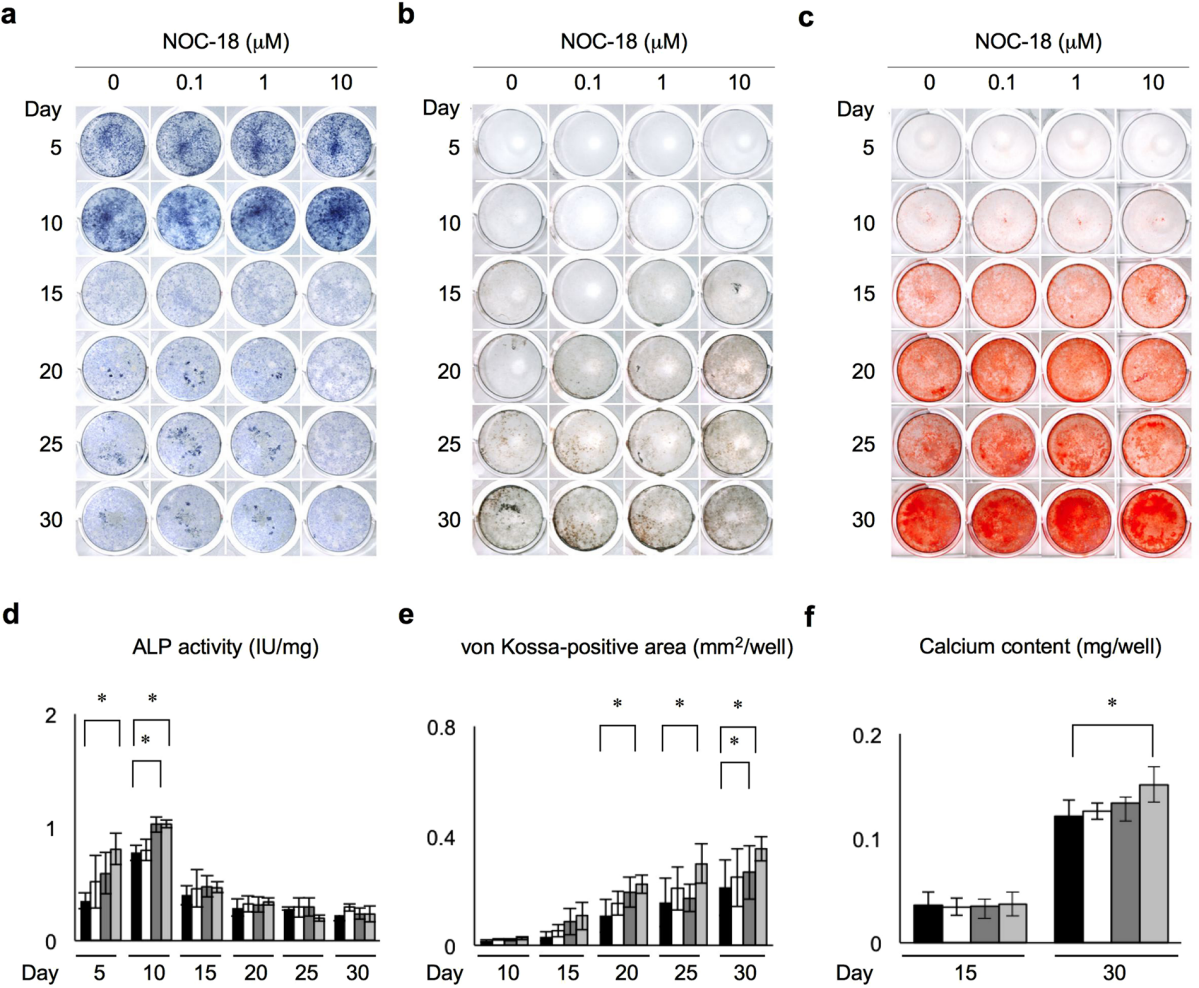
Effects of NOC-18 on the odontogenic capacity of rDPSCs. (**a,d**) Effects of NOC-18 on alkaline phosphatase (ALP) activity as assessed by ALP staining (**a**) and ALP activity test (**d**). (**b,e**) Effects of NOC-18 on phosphate deposition by von Kossa staining (**b**) and measurement of the von Kossa-positive area (**e**). (**c,f**) Effects of NOC-18 on calcium deposition by Alizarin Red-S staining (**c**) and measurement of calcium content (**f**). (**d,e,f**) n = 3 per group; *p < 0.05. Graph bars are the means ± SEM.

To investigate the effects of exogenous NO on the odontoblast differentiation and dentin formation of rDPSCs, these cells were cultured under odontogenic-inducing conditions in the presence of 0, 0.1, 1.0, and 10 μM NOC-18. An ALP activity assay demonstrated that ALP activity was enhanced in rDPSCs at all tested concentrations of NOC-18 (Fig. 2a,d). In NOC-18-treated cells ALP activity increased beginning at day 5, especially at 10 μM NOC-18, and reached a maximum on day 10, especially at 1 and 10 μM NOC-18. On day 15, ALP activity was remarkably reduced in all NOC-18-treated groups, and gradually decreased until the end of the experimental culture period, on day 30.

Alizarin red-S and von Kossa staining assays showed that mineralized nodule formation was enhanced in both a time- and dose-dependent manner under NOC-18 stimulation (Fig. 2b,c,e, and f). In particular, the von Kossa-positive area was significantly increased in the presence of 10 μM NOC-18 after day 20, and especially on day 30 (Fig. 2e). In addition, Alizarin Red-S staining showed significantly increased calcium accumulation in the nodules on day 30 at all tested concentrations of NOC-18, especially 10 μM NOC-18 (Fig. 2f).

**Figure 3:**
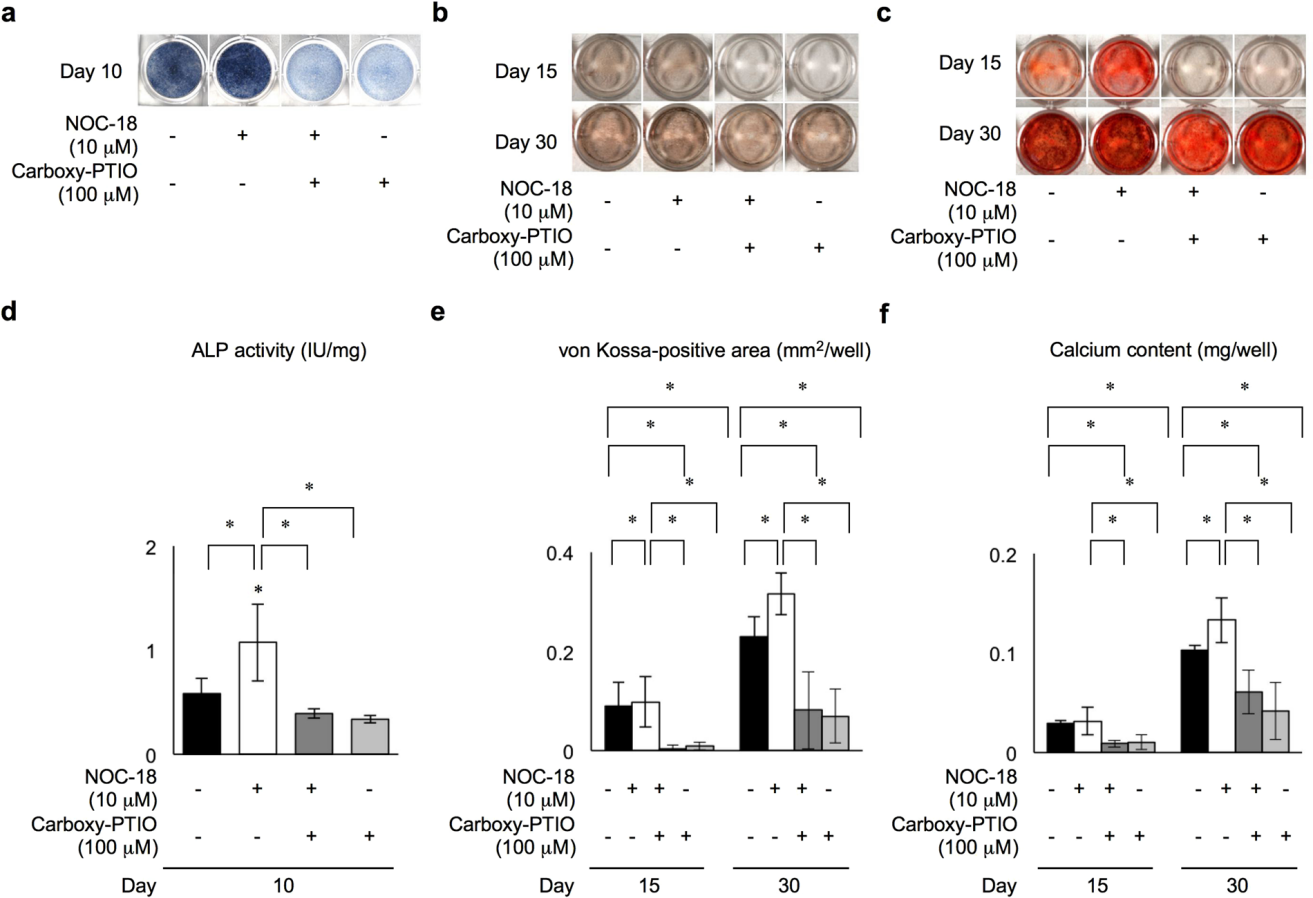
Effects of the NO scavenger carboxy-PTIO on the odontogenic capacity of rDPSCs under stimulation with NOC-18. (**a,d**) Effects of carboxy-PTIO on ALP activity by ALP staining (**a**) and ALP activity test (**d**). (**b,e**) Effects of carboxy-PTIO on phosphate deposition by von Kossa staining (**b**) and measurement of the von Kossa-positive area (**e**). (**c,f**) Effects of carboxy-PTIO on calcium deposition by Alizarin Red-S staining (**c**) and measurement of calcium contents (**f**). (**d,e,f**) n = 3 per group; *p < 0.05. Graph bars are the means ± SEM.

To further confirm the effects of NOC-18 on the mineralized tissue formation capacity of rDPSCs *in vitro*, these cells were treated with 100 μM carboxy-PTIO in the presence of 10 μM NOC-18. On day 10, the ALP activity in NOC-18-treated and untreated rDPSCs was significantly reduced by carboxy-PTIO-treatment under odontogenic induction conditions (Fig. 3a,d). In addition, the von Kossa-positive and Alizarin Red-S-positive nodules were markedly reduced in rDPSCs treated with carboxy-PTIO compared with these positive nodules in control rDPSCs without carboxy-PTIO treatment under odontogenic induction conditions (Fig. 3b,c,e,f). The effects of Carboxy-PTIO on NO elimination in rDPSC culture were confirmed before the odontogenic induction test (Supplementary Figure 3).

### NOC-18 stimulates the expression of odontoblast phenotype-related genes in DPSCs

**Figure 4:**
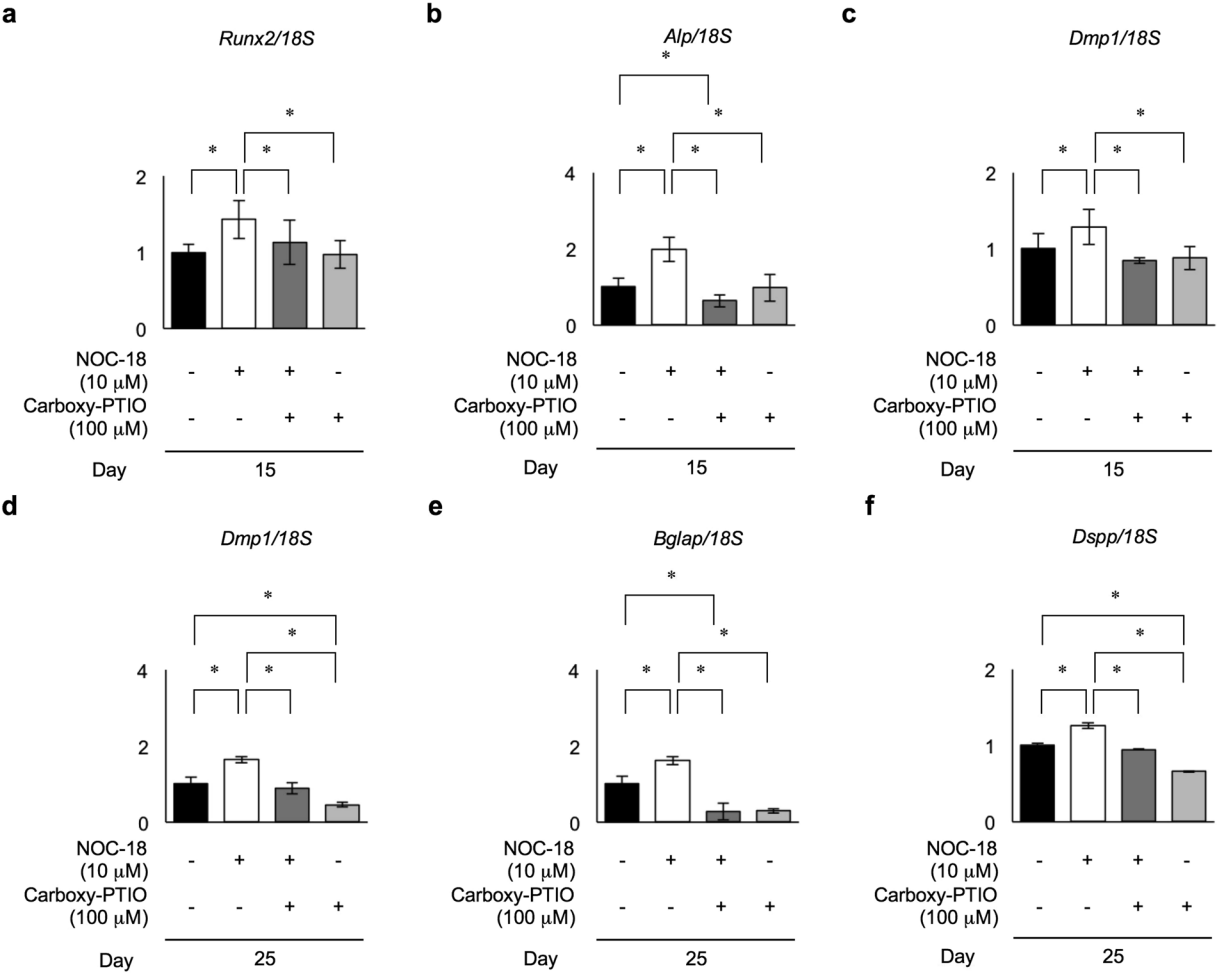
Effects of NOC-18 and carboxy-PTIO on the expression of odontoblast-specific genes in rDPSCs. Expression of odontoblast-specific genes by real-time RT-PCR at the early (day 15) (**a–c**) and late (day 25) (**d–f**) stages of differentiation. Expression of runt-related transcription factor 2 *Runx2* (**a**), *Alp* (**b**), dentin matrix protein 1 (*Dmp1*) (**c,d**), bone gamma-carboxyglutamic acid-containinig protein (*Bglap*) (**e**), and dentin sialophosphoprotein (*Dspp*) (**f**). (**a–f**) n = 3 per group; *p < 0.05. Each gene was normalized to 18S ribosomal RNA (18*S*) in each sample. Graph bars are the means ± SEM.

To investigate the effects of exogenous NO during odontoblast differentiation of rDPSCs on a molecular level, we examined the expression of odontoblast-specific genes in rDPSCs at the early (day 15) and late (day 25) stages of odontogenic differentiation in the presence of 10 μM NOC-18 by real-time RT-PCR. Early stage-specific genes, including runt-related transcription factor 2 (*Runx2*), type I collagen, *Alp*, bone sialoprotein, and dentin matrix protein 1 (*Dmp1*), were significantly higher in NOC-18-treated rDPSCs than in untreated control cells (Fig. 4a–c, Supplementary Figure 2). Late stage-specific genes, including *Dmp1*, bone gamma-carboxyglutamic acid-containinig protein (*Bglap*), and *Dspp*, were also significantly upregulated in NOC-18-treated cells (Fig. 4d–f). To evaluate the effects of NOC-18 on gene expression in rDPSCs, these cells were treated with 100 μM carboxy-PTIO in the presence or absence of 10 μM NOC-18 under odontogenic-inducing conditions. Carboxy-PTIO treatment markedly reduced the enhanced expression of these genes in rDPSCs under NOC-18 stimulation at both the early and late stages of odontogenic differentiation, even in the presence of Carboxy-PTIO without NOC-18 stimulation (Supplementary Figures 2–4). These findings suggested that exogenous NO, as well as endogenous NO, induced the odontoblast differentiation capacity of rDPSCs.

### NOC-18 stimulates iNOS expression and NO production in DPSCs

**Figure 5:**
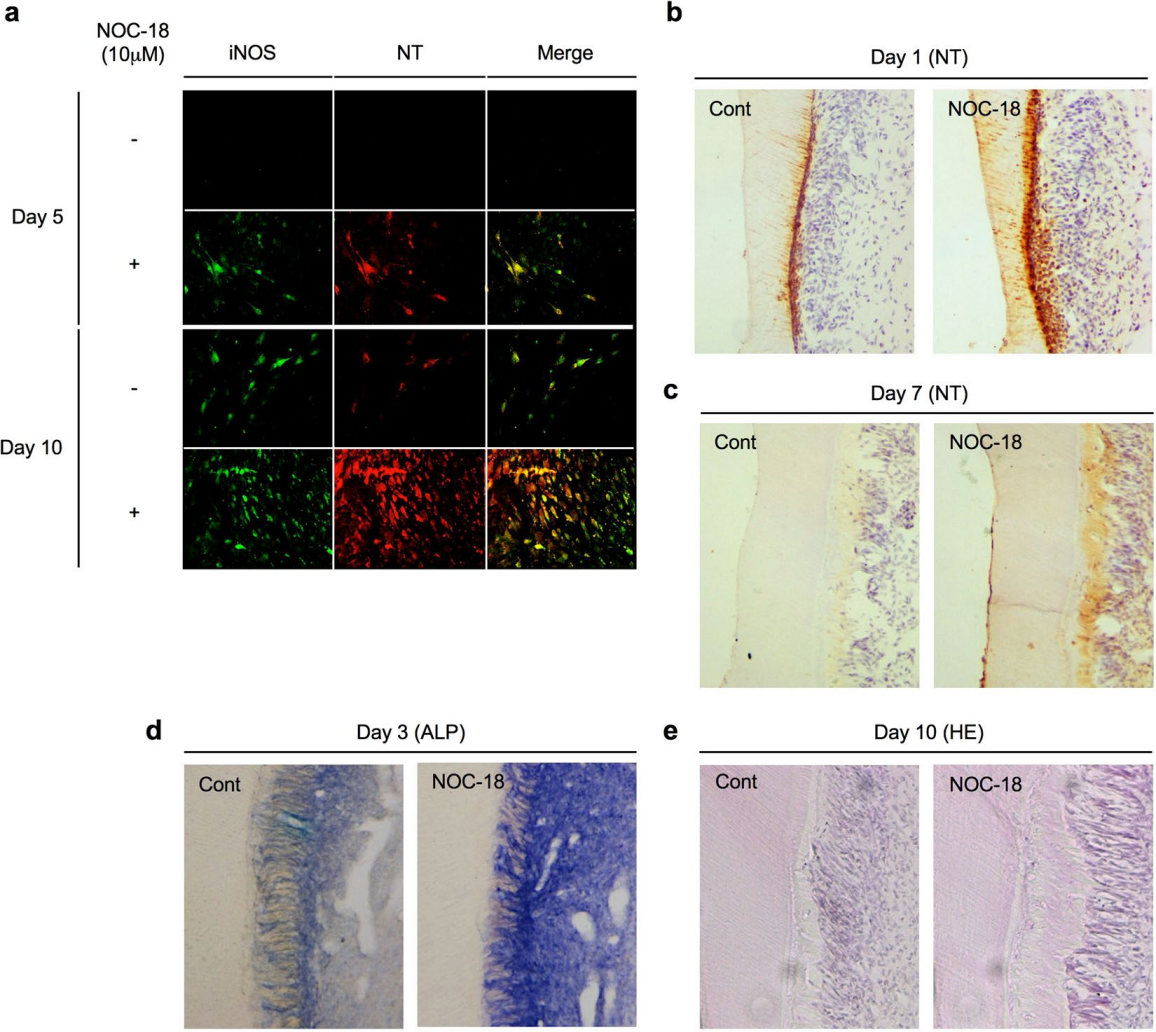
Effects of NOC-18 on iNOS expression, NO production, and tertiary dentin formation *in vivo*. (**a**) Expression of inducible nitric oxide synthase (iNOS) and nitrotyrosine (NT) in rDPSCs treated with NOC18 (10 μM) as assessed by double immunofluorescence. Merge: Merged images of iNOS and NT. (**b–e**) Effects of NOC-18 on tertiary dentin formation in the dentin-dental pulp complex under a tooth cavity. Localization of NT on days 1 (**b**) and 7 after tooth preparation by immunohistochemistry (**c**). ALP activity on day 3 by immunohistochemistry (**d**). Tertiary dentin formation on day 10 by hematoxylin and eosin (HE) staining (**e**).

To examine the effects of NOC-18 on NO production in odontoblast differentiation, we investigated the expression of a strong NO synthase, iNOS, and one of the critical markers of NO-mediated tyrosine products, nitrotyrosine, in rDPSCs stimulated with or without 10 μM NOC-18 under odontogenic-inducing conditions by double immunofluorescent staining. The expression of iNOS and nitrotyrosine was increased in NOC-18-treated rDPSCs on days 5 and 10 after odontogenic induction (Fig. 5a), but decreased on day 30 (Data not shown). In contrast, iNOS and nitrotyrosine expression levels in untreated rDPSCs (without NOC-18) were low on day 10 after odontogenic induction (Fig. 5a). These findings suggest that exogenous NO can induces iNOS expression and abundant NO release in immatured rDPSC-derived odontogenic cells at the early stage of odontogenic differentiation. However, because abundant mineralized nodules were occupied on the culture well surface on day 30, iNOS and nitrotyrosine expression might be disappeared in the matured odontogenic cells.

### NOC-18-enhanced TNFα-NF-κB axis accelerates odontogenic differentiation in DPSCs

**Figure 6:**
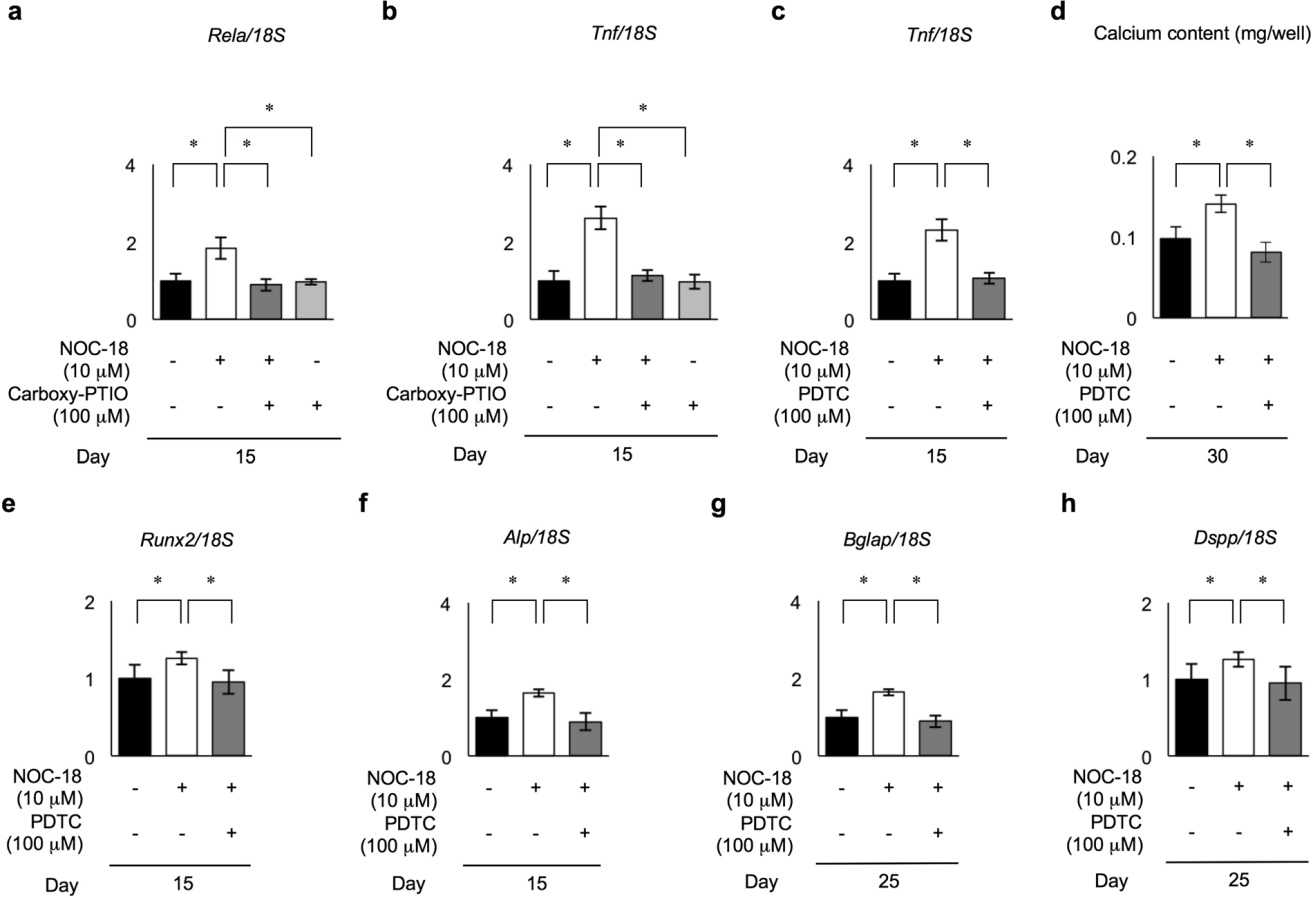
Effects of NOC-18-induced Rela (NF-kB p65) on odontogenic capacity of rDPSCs. (**a,b**) Expression of *Rela* (**a**) and tumor necrosis factor alpha (*Tnf*) (**b**) genes by real-time RT-PCR at the early (day 15) stages of the odontogenic differentiation. (**c**) Effect of NF-κB inhibitor PDCT (100 μM) on *Tnf* expression in rDPSCs at the early (day 15) stages of the odontogenic differentiation by real-time RT-PCR. (**d–h**) Effect of NF-κB inhibitor PDCT (100 μM) on the odontogenic differentiation of rDPSCs by real-time RT-PCR. Measurement of calcium contents by Alizarin Red-S staining (**d**). Expression of *Runx2* (**e**) and *Alp* (**f**) at the early (day 15) stage and *Bglap* (**g**) and *Dspp* (**h**) at the late (day 25) stage. (**a–h**) n = 3 per group; *p < 0.05. Graph bars are the means ± SEM. (**a–c,e–h**) Each gene was normalized to 18S ribosomal RNA (*18S*) in each sample.

NF-κB activation plays a critical role in expression of iNOS via a variety of cytokines such as tumor necrosis factor alpha (TNFα)^5^. In this study, NOC-18 induced iNOS expression in rDPSCs during the dentinogenic induction, especially at the early stage. Therefore, we hypothesized that NF-κB might play a role in producing abundant NO via iNOS in NOC-18-treated DPSCs. Real-time RT-PCR assay analyzed the expression of *Rela* and *Tnf* in rDPSCs under odontogenic condition in the presence or absence of NOC-18 (10 μM) and carboxy-PTIO (100 μM). On day 15, *Rela* and *Tnf* were significantly upregurated in NOC-18-treated rDPSCs compared to the untreated control cells (Fig. 6a,b). On the other hands, carboxy-PTIO-treated group reduced the enhanced expression of *Rela* and *Tnf* under NOC-18 treatment, but not under NOC-18 untreatment (Fig. 6a,b). To comfirm the participant of NF-κB pathway in NOC-18 accelerated odontogenic differentiation of DPSCs, rDPSCs were treated with a NF-κB inhibitor PDTC (100 μM). PDTC treatment reduced the enhanced expression of *Tnf* in NOC-18 stimulated rDPSCs (Fig. 6c). Alizarin Red-S staining on day 30 showed PDTC treatment significantly supressed the increased calcium accumulation in the nodules in NOC-18-treated rDPSCs (Fig. 6d). PDTC treatment also reduced the enhanced odontoblast-specific genes, including both the early stage-specific genes *Runx2* and *Alp* and both the late stage-specific genes *Bglap* and *Dspp*, under NOC-18-treated rDPSCs (Fig. 6e–h).

### NOC-18 induces tertiary dentin formation *in vivo*

Recent studies demonstrated that newly-recruited odontoblast precursors/newly differentiated odontoblast-like cells, which could be recruited from DPSCs in the perivascular niche of dental pulp tissues^4^, were located in the dental pulp tissues just beneath the cut dentin 1 day after tooth preparation, and these cells subsequently form the tertiary dentin^13,14^. In the newly-recruited odontoblast precursor cells/newly-differentiated odontoblast-like cells, iNOS and nitrotyrosine were temporarily expressed on 1 day after tooth preparation. In this study, we asked whether NOC-18 treatment induced the formation of tertiary dentin in a rat model. Immunohistochemical analysis demonstrated that, on day 1 after tooth preparation, the expression of nitrotyrosine was higher in the cell population in the dental pulp tissues and dentinal tubules beneath NOC-18-treated cavities than in the tissues beneath non-treated cavities (Fig. 5b). Nitrotyrosine immunoreactivity under both treatment cavities disappeared on day 7 (Fig. 5c). On day 3, ALP activity was markedly induced in the dental pulp tissues beneath NOC-18-treated cavities compared to the activity levels beneath untreated cavities (Fig. 5d). Histochemical analysis showed that a larger amount of tertiary dentin was formed beneath the dentin of NOC-18-treated cavities than beneath untreated cavities on day 10 (Fig. 5e).

## Discussion

Because DPSCs exhibit strong capacities for rapid cell proliferation and odontoblast differentiation^2^, they are a strong candidate for reparative reconstruction of tertiary dentin under the condition of reversible pulpitis after strong noxious injuries. Recent *in vitro* osteoblast-like cell culture experiments showed that exogenous NO promoted osteogenic differentiation, which was associated with the expression of ALP and BGLAP and the formation of mineralized tissue in osteoblasts^7,15,16^. The results of the present *in vitro* study using the NO donor NOC-18 and NO scavenger carboxy-PTIO indicated that NOC-18-released exogenous NO stimulated the odontoblast-specific genes *Runx2*, *Alp*, *Dmp1*, *Bglap*, and *DSPP* and mineralized tissue formation in rDPSCs. Interestingly, the results also indicated that NOC-18 induced a temporary increase in iNOS and nitrotyrosine production in rDPSCs *in vitro* and rDPSC-derived odontoblast-lineage cells *in vivo*, suggesting that exogenous NO could strongly commit DPSCs to mature odontoblasts at the early stage of the differentiation process. Further *in vivo* investigation with an experimental tooth preparation model in rats showed that tertiary dentin formation was actively induced by NOC-18, suggesting that exogenous NO might be effective for treating damaged dental pulp tissues.

Many genes encoding transcription factors and matrix proteins involved in differentiation are expressed in both odontoblasts and osteoblasts^17,18^. In addition, more than 4,000 genes expressed in human DPSCs are also expressed in human bone marrow MSCs^19^. *RUNX2*, *DSPP*, and *DMP1* are expressed in DPSCs during odontogenic differentiation^2,20^. Runx2, which was first identified as a crucial transcription factor in osteoblasts^21^, suppresses the expression of an odontoblast terminal marker, DSPP^22^. DMP1, which is a major non-collagenous protein in the dentin matrix^23^, functions as a transcriptional factor to promote the expression of DSPP at the terminal stage of odontoblast differentiation^24^. *Dmp1* is transcriptionally regulated by the AP-1 components, such as c-Jun, JunB, and c-Fos^25,26^. During the differentiation of osteoblast-like cells, exogenous NO-induced cyclic guanosine 3′,5′-monophosphate (cGMP) activates a c-Fos promoter element^7,16,27^. In our *in vitro* donor/scavenger experiments, NOC-18-released exogenous NO increased the expression of *Runx2* and *Dspp* at the early and late stages, respectively, of odontogenic differentiation of rDPSCs. Exogenous NO also induced the expression of *Dmp1* at the early and late stages of odontogenic differentiation, suggesting that exogenous NO regulates the expression of stage-specific genes, such as *Runx2*, *Dmp1*, and *Dspp*, in DPSCs to commit them to mature odontoblasts.

Generally, NF-κB activation induced iNOS expression via a variety of cytokines such as TNFα in cells^5^. In this study, NOC-18 induced iNOS expression during the dentinogenic induction, especially at the early stage, suggesting that NF-κB might play a role in producing abundant NO via iNOS in NOC-18-treated DPSCs. The present real-time RT-PCR assay demonstrated that NOC-18 stimulated the expression of *Rela* in rDPSCs. NF-κB-specific inhibitor PDTC suppressed NOC-18-enhanced odontogenic differentiation associated with calcium accumulation and odontoblast-specific gene expression including *Runx2*, *Alp*, *Bglap* and *Dspp*. PDTC also inhibited the increased expression of *Tnf* in odontogenic DPSCs stimulated with NOC-18. Recent study demonstrates that TNFα accelerates odontogenic differentiation of DPSCs through NF-κB pathway^28^. These findings suggest that exogenous NO-released TNFα in DPSCs induces the odontogenic differentiation of DPSCs via NF-κB pathway.

Vital pulp therapy is used in the treatment of reversible pulpitis to maintain the viability and function of the injured dental pulp, and two therapeutic approaches, indirect and direct pulp capping, are used for deep dentinal cavities and pulp exposure, respectively. Recent advances in stem cell biology in the dental field have suggested a novel endodontic approach to regenerate the dental-pulp complex^29,30^. The ultimate goal of using a regenerative strategy for vital pulp therapy with direct pulp capping is to reconstruct the normal dentin-pulp structure at the lesion under deep caries^31^. Our novel approach is based on an understanding of the molecular and cellular mechanisms regulating the tissue-specific repair process of tertiary dentin formation by endogenous (host) dental pulp tissue-specific stem cells. To achieve this therapeutic goal, we need to develop an alternative to the current indirect capping agents. Recent studies of experimental tertiary dentin formation demonstrated that a DMP1-capping treatment induced the cytodifferentiation of DPSCs into odontoblasts that participate in the formation of tertiary dentin^32,33^. The present *in vivo* results indicated that NOC-18 induced the formation of newly differentiated odontoblasts or odontoblast precursor cells by recruiting host DPSCs from the perivascular niche to the lesion under the prepared cavity. Likewise, the present *in vitro* results showed that NOC-18-released NO induced odontoblast-specific genes, such as *Runx2*, *Dmp1*, and *Dspp*, and activated tertiary dentin formation in DPSCs. Therefore, our findings suggest a model in which NOC-18 in the cavity releases gas-formed NO into the pulp lesion through the dentinal tubules to recruit and stimulate DPSCs from the perivascular niche into the dental pulp^4^ (Supplementary Figure 4), suggesting NOC-18 as a novel pulp-capping agent for use in endodontics.

In conclusion, the present findings, using a NO donor and scavenger, indicate that exogenous NO induces DPSCs via TNFα-NF-κB axis to differentiate into mature odontoblasts, which was associated with the expression of stage-specific genes, such as *Dmp1* and *Dspp*, but did not affect viability, suggesting the NO releasing donor NOC-18 as an alternative to current pulp capping agents for endodontic treatment. Further studies are needed to determine the detailed molecular mechanisms underlying the function of NOC-18-released exogenous NO in the odontogenic differentiation of DPSCs as well as experimental evaluations in pre-clinical studies and clinical trials for regenerative endodontics.

## Methods

### Animals

Wistar rats were purchased from CLEA Japan (Shizuoka, Japan). All animal experiments were performed according to protocols approved by the Institutional Animal Care and Use Committee of Kyushu University (Protocol Number: A21-044-1).

### Isolation and culture of rat dental pulp stem cells (rDPSCs)

Rat DPSCs (rDPSCs) were isolated from the incisor dental pulp tissues of neonatal 1 day-old rats as previously described^34^. The mandibular incisors were aseptically removed from the mandibular bones, and the pulp tissues were extracted and cut into small pieces. The obtained tissue fragments were incubated with 0.2% collagenase type I (Worthington Biochemicals, Lakewood, NJ) and 0.4% dispase II (Sanko Junyaku, Tokyo, Japan) for 60 min at 37 °C, and then passed through a 70-μm cell strainer to obtain a single-cell suspension. The cells were seeded in 100-mm culture dishes and were incubated for 3 hours at 37 °C to allow cell attachment. The cultures were then rinsed twice with sterilized phosphate-buffered saline (PBS) to remove non-adherent cells. The remaining adherent cells were incubated in alpha-modified minimum essential medium (αMEM; Thermo Fisher Scientific, Waltham, MA) supplemented with 20% fetal bovine serum (FBS; Equitech-Bio, Kerrville, TX), 100 µM L-ascorbic acid 2-phosphate (Wako Pure Chemicals, Osaka, Japan), 2 mM L-glutamine (Nacalai Tesque, Kyoto, Japan), 55 mM 2-mercaptoethanol (2-ME; Thermo Fisher Scientific), and penicillin/streptomycin (100 U/mL penicillin and 100 g/mL streptomycin; Nacalai Tesque) at 37 °C and 5% CO_2_. Adherent colony-forming cells observed 14 days after seeding were considered to be passage 0 (P0) rDPSCs. These cells were passaged once, to P1 cells, and were maintained in the above mentioned growth medium. The medium was changed twice a week. P1 cells were generally used for experiments. A detailed characterization of the P1 cells, according to the method in a previous study^34^, is described in the Supplementary Methods.

### Treatment with a nitric oxide (NO) donor and scavenger

rDPSCs P1 cultures were treated with the NO donor NOC-18 (0, 0.1, 1.0, and 10 μM; Dojindo Laboratories, Kumamoto, Japan) and/or the NO scavenger carboxy-PTIO (100 μM; Dojindo Laboratories) and were used for the experiments described below.

### *In vitro* odontogenic culture of rDPSCs

rDPSCs were cultured in 24-well multiplates or 35-mm culture dishes in odontogenic medium consisting of alpha-minimum essential medium (Thermo Fisher Scientific) supplemented with 20% FBS (Equitech-Bio), 2 mM β-glycerophosphate (Sigma-Aldrich, St Louis, MO), 100 mM L-ascorbic acid 2-phosphate (Wako Pure Chemicals), 10 nM dexamethasone (Sigma-Aldrich), 2 mM L-glutamine (Nacalai Tesque), 55 μM 2-ME (Thermo Fisher Scientific), and penicillin/streptomycin (Nacalai Tesque). The medium was changed twice a week. To block NF-kB pathway, rDPSCs were treated with 100 μM pyrrolidine dithocarbamate (PDTC, Sigma-Aldrich), according to the previous study^28^.

### Alkaline phosphatase (ALP) bioactivity assay

ALP bioactivity was examined by ALP staining and ALP activity testing according to previously described methods^35^. For ALP staining, rDPSCs cultured in 24-well multiplates were fixed with 10% formalin for 10 min. Then, the cells were stained with 0.1% naphthol AS-MX phosphate (Sigma-Aldrich) and 0.1% fast blue BB salt (Sigma-Aldrich) in 0.1 M Tris-buffer (pH 9.2) at 37 °C for 30 min, and then washed with distilled water. For the ALP activity test, rDPSCs were cultured in 35-mm dishes for predetermined time periods, and ALP activity was assayed using the ALP IB test kit (Wako Pure Chemicals) according to the manufacturer’s instructions. The absorbance at 405 nm was measured with a Multiscan GO spectrometer (Thermo Fisher Scientific). Total cellular proteins were determined using the BCA protein assay (Thermo Fisher Scientific) by measuring the absorbance at 595 nm. ALP activity values were normalized to the relative concentrations of total cellular protein.

### Mineralization assays

Mineralization assays of rDPSCs cultured in 24-well multiplates were performed as described previously^35^. For Alizarin red S staining, the cultures were fixed in 10% formalin and incubated with 1% Alizarin red S (Sigma-Aldrich) for 10 min at room temperature. For von Kossa staining, cultures were treated with 5% silver nitrate (Sigma-Aldrich) for 60 min at room temperature, and then with 3% sodium thiosulfate (Nacalai Tesque) for 3 min. Finally, all of the cultures were washed in distilled water. The total area of von Kossa-positive nodules was analyzed with MCID image analysis software (Image Research, Inc., Brock University, Ontario, Canada). To measure calcium content in the mineralized nodules, the cultures were treated with 0.1 M HCl overnight, and then calcium content was assessed with the Calcium C-test kit (Wako Pure Chemicals) according to the manufacturer’s instructions and measured at 570 nm with a Multiscan GO spectrometer (Thermo Fisher Scientific).

### Gene expression assay

Total RNA was extracted from cultured cells with TRIzol (Thermo Fisher Scientific), digested with DNase I (Promega, Madison, WI), and purified using the RNeasy Mini kit (Qiagen, Venlo, Netherlands). Then, purified total RNA (1 µg) was reverse transcribed with the ReverTra Ace qPCR kit (TOYOBO, Osaka, Japan). For the qRT-PCR assay, cDNA was amplified using TaqMan Gene Expression Master Mix (Applied Biosystems, Foster City, CA) and target TaqMan probes (Applied Biosystems) with a Light Cycler 96 real-time PCR system (Roche, Basel, Switzerland). The 18S ribosomal RNA gene was used for normalization. All of the probes used are summarized in Supplementary Table 1.

### Double immunofluorescence

P1 rDPSCs pretreated with 10% normal goat serum (Vector Laboratories, Burlingame, CA) were incubated with anti-nitrotyrosine rabbit IgG (1:100; Millipore, Billerica, MA) at 4 °C overnight, and then incubated with goat biotinylated anti-rabbit IgG (1:200; Vector Laboratories) for 60 min. After treatment with 10% non-immune goat serum for 30 min, the samples were incubated with anti-iNOS rabbit IgG (1:100; Santa Cruz Biotechnology, Santa Cruz, CA) at 4 °C overnight. Then, they were incubated with fluorescein-labeled goat anti-rabbit IgG (1:50; Thermo Fisher Scientific) and Texas Red-conjugated streptavidin (1:100; Vector Laboratories) for 60 min. The immunohistochemical specificity of these antibodies was evaluated in previous studies^14,36,37^. Finally, after washing, sections were mounted in VECTASTAIN anti-fade medium with 4ʹ,6-diamidino-2-phenylindole (DAPI; Vector Laboratories). The sections were observed under an Axio Imager M2 microscope (Carl Zeiss Microscopy, Oberkochen, Germany).

### Tooth preparation model

A tooth cavity was created on the mesial surface of the maxillary first molars of Wistar rats (male, 4-weeks old) with a low-speed micro-motor hand-piece (C-150; MINITOR, Tokyo, Japan) equipped with an inverted cone bur (No. 33 1/2; Dentsply, York, PA) under continuous sterile saline, as previously described^13,14^. The cavities were wiped with absorbent cotton, and treated with or without NOC-18 (100 μM). Finally, the cavities were filled with a glass ionomer cement (Fuji ionomer type II; GC Corporation, Tokyo, Japan).

### Tissue preparation

At the conclusion of the experimental period, the animals were perfused with ice-cold 4% paraformaldehyde and 0.2% saturated picric acid in 0.1 M PBS, pH 7.4. Maxillae-containing tooth samples were removed *en bloc* and immersed in fresh fixative for 3 hours at 4 °C. Then, the samples were decalcified with 4% EDTA in 0.01 M PBS, pH 7.4, at 4 °C for 10 days, and immersed in 0.1 M PB, pH 7.4, containing 20% sucrose at 4 °C. The tissues were then were embedded in OTC compound (Sakura, Tokyo, Japan) and frozen in dry-ice/isopentane. The embedded tissues were cut into 10-μm-thick sections on a CM1950 cryostat (Leica, Solms, Germany).

### Histochemical assays

Tissue sections were stained with hematoxylin and eosin. For ALP activity staining, the sections were stained with 0.1% naphthol AS-MX phosphate (Sigma-Aldrich) and 0.1% fast blue BB salt (Sigma-Aldrich) in 0.1 M Tris-buffer (pH 9.2) at 37 °C for 30 min^14^. Immunohistochemistry with anti-nitrotyrosine antibody was performed as described previously^14^. The sections were quenched with 0.3% H_2_O_2_ in PBS for 60 min and blocked with 10% non-immune goat serum (Vector Laboratories) for 30 min. Then, the samples were incubated with anti-nitrotyrosine antibody (1:100; Millipore) at 4 °C overnight. Next, the sections were incubated with a biotinylated antibody (1:200; Vector Laboratories) for 45 min, followed by avidin-biotin complex (1:100; Vector Laboratories) for 60 min. The sections were reacted with 0.02% 3,3″-diaminobenzidine tetrahydrochloride (Dojindo Laboratories) and 0.06% H_2_O_2_ in 0.05 M Tris buffer, pH 7.6 and counter-stained with hematoxylin. Immunohistochemical controls were incubated with non-immune rabbit IgG (Vector Laboratories) instead of the primary antibody. The sections were observed under an Axio Imager M2 microscope.

### Statistics

All data are expressed as the mean and standard error of the mean (SEM) or standard deviation (SD) of at least three determinations. Comparisons between two groups were analyzed by independent two-tailed Student’s t-tests. Multi-group comparisons were analyzed by one-way repeated measures analysis of variance followed by Tukey’s post hoc test. P values less than 0.05 were considered statistically significant.

## Electronic supplementary material

Supplementary Information
