# Supplementary material for: Exogenous nitric oxide stimulates the odontogenic differentiation of rat dental pulp stem cells
Source: Sci Rep. 2018 Feb 21;8:3419. doi: 10.1038/s41598-018-21183-6 (PMC5821879; doi:10.1038/s41598-018-21183-6)
Supplement: Supplementary file 1 — Supplementary Information [file 41598_2018_21183_MOESM1_ESM.pdf]

Supplementary Information

Exogenous nitric oxide stimulates the odontogenic differentiation of rat dental pulp stem cells

Soichiro Sonoda, Yu-feng Mei, Ikiru Atsuta, Atsushi Danjo, Haruyoshi Yamaza, Shion Hama, Kento Nishida, Ronghao Tang, Yukari Nakamura-Kyumoto, Norihisa Uehara, Toshio Kukita, Fusanori Nishimura, Takayoshi Yamaza

Supplementary Methods

Colony-forming units-fibroblastic (CFU-F) assay

CFU-F assay was performed as described previously (Yamaza et al. 2011). Briefly, isolated cells from rat dental pulp tissues were seeded at 1-10x10^3 per dish on 60 mm culture dishes. Fourteen days after the seeding, the cultures were treated with a mixture of 2% paraformaldehyde and 0.1% toluidine blue. Number of colonies containing 50 cells < cells were counted with Axio Imager M2 microscope (Carl Zeiss Microscopy, Jena, Germany).

Bromo-deoxyuridine (BrdU) incorporation assay

rDPSCs (P1, 1x10^3) on 35-mm dishes were treated with BrdU reagent (1:100, Thermo Fisher Scientific, Waltham, MA) for 24 hours. BrdU-positive cells were detected by using BrdU staining kit (Thermo Fisher Scientific). BrdU-positive nuclei were observed with Axio Imager M2 microscope (Carl Zeiss Microscopy), and were quantified according to the previous report (Yamaza et al. 2010).

Flow cytometric analysis

rDPSCs (P1) at 50-60% confluent were removed. The cells (0.1x10^6) were stained with R-phycoerythrin-conjugated anti-human CD29, CD45 and CD90 antibodies (each 1 μg) (BioLegend, San Diego, CA) in 100 μl of flow cytometry buffer containing 2% heat-activated FBS (Equitech-Bio, Kerrville, TX) in PBS for 45 min on ice. The stained cells were washed for 5 min at 3 times, and were measured on FACSVerse flow cytometer (BD Bioscience, Franklin Lake, NJ). The sub-class-matched antibodies (BioLegend) were used as the controls. The number

(percentage) of positive cells in $10x10^3$ was determined using FACSuite software (BD Bioscience) in comparison with the corresponding control cells stained with corresponding sublass-matched antibody in which a false-positive rate of less than 1% was accepted (Yamaza et al. 2010).

Multi-differentiation assay

***in vitro* odontogenic assay**. rDPSCs (P1, $3x10^5$) were seeded on 35-mm dishes, and cultured until they reached confluence, and were induced in an osteogenic medium. The osteogenic medium was consisted of αMEM (Thermo Fisher Scientific) containing 20% FBS (Equitech-Bio), 2 mM β-glycerophosphate (Sigma-Aldrich, St Louis, MO), 100 mM L-ascorbic acid 2-phosphate (Wako Pure Chemicals, Osaka, Japan), 10 nM dexamethasone (Dex) (Sigma-Aldrich), 2 mM L-glutamine (Nacalai Tesque, Kyoto, Japan), 55 μM 2-ME, and penicillin/streptomycin-mixed antibiotics (Nacalai Tesque) for 6 weeks. For mineralized nodule detection, the cultures were stained with 1% Alizarin red-S (Sigma-Aldrich) in distilled water, and observed with Primovert microscope (Carl Zeiss Microsocopy).

***in vitro* chondrogenic assay**. For chondrogenic differentiation, cell pellets of rDPSCs (P1, $1.0x10^6$) were cultured with a chondrogenic medium containing Dulbecco's Modified Eagle Medium (Thermo Fisher Scientific) with 15% FBS (Equitech-Bio), 2 mM L-glutamine (Nakalai Tesque), 1% insulin-transferrin-selenium (ITS) (Corning, Corning, NY), 100 nM dexamethasone (Sigma-Aldrich), 100 mM L-ascorbic acid 2-phosphate (Wako Pure Chemicals), 2 mM sodium pyruvate (Nakalai Tesque), 1 ng/ml TGF-β_3 (Peprotech, Rocky Hill, NJ), and penicillin/streptomycin-mixed antibiotics (Nacalai Tesque). Four weeks after the induction, the samples were fixed with 4% paraformaldehyde in PBS, pH 7.4. The paraffin sections were cut, and treated with alcian blue staining using alcian blue staining solution (Poly Scientific, Bay Shore, NY). The sections were observed with Axio Imager M2 microscope (Carl Zeiss Microscopy).

***in vitro* adipogenic assay.** P1 MSCs were cultured in an adipogenic inductive medium containing αMEM (Thermo Fisher Scientific) with 15% FBS (Equitech-Bio), 2 mM L-glutamine (Nakalai Tesque), 0.5 mM isobutyl-methylxanthine (Sigma-Aldrich), 60 mM indomethacin (Sigma-Aldrich), 0.5 mM hydrocortisone (Sigma-Aldrich), 10 mM insulin (Sigma-Aldrich), and 100 U/ml penicillin and

penicillin/streptomycin-mixed antibiotics (Nakalai Tesque). Six weeks after the induction, they were fixed with 4% paraformaldehyde in PBS for 10 min, and stained for lipid droplet with 0.3% Oil red O (Sigma-Aldrich) in isopropanol. The cultures were observed with Primovert microscope (Carl Zeiss Microsocpy).

Cell viability assay

rDPSCs (P1) were cultured on 96-well multiplates for 1, 2, 3, or 5 days with or without NOC-18 (10 μM) (Dojindo Laboratories, Kumamoto, Japan) and carboxy-PTIO (100 μM) (Dojindo Laboratories). The cell viability was examined using WST-8 cell count kit (Dojindo Laboratories) according to the manufacturer's instructions, and was measured at 450 nm with Multiscan GO spctrophotometer (Thermo Fisher Scientific).

Morphology

rDPSCs (P1) were cultured in the presence or absence of NOC-18 (10 μM) (Dojindo Laboratories) for 3 days, and were fixed with 4% paraformaldehyde in 0.1 M phosphate buffer (PB), pH 7.4, for 15 min at room temperature. They were incubated with periodate (4.2 mM) (Nacalai Tesque) for 30 min at 4°C, and were stained with fluorescein-5-thiosemicarbazide (0.1 mM) (Thermo Fisher Scientific) at 37°C for 120 min. The cells were observed with Zeiss Axio Imager M2 microscope (Carl Zeiss Microscopy).

Measurement of NO_2^- in conditioned medium

NO was measured as NO_2^- products in the conditioned medium of rDPSC cultures. rDPSCs (P1) were cultured on 24-well multiplates for 48 h with or without NOC-18 (10 μM) (Dojindo Laboratories) and carboxy-PTIO (100 μM) (Dojindo Laboratories). The conditioned mediums were collected and used for measuring NO_2^- using NO2/NO3 Assay Kit-CII (Colorimetric) ~Geiss Reagent Kit~ (Dojindo Laboratories) at 540 nm with Multiscan GO spctrophotometer (Thermo Fisher Scientific). Nitrite level was normalized with protein amount, which was measured using Protein assay (Bio-Rad, Hercules, CA) at 595 nm with Multiscan GO spctrophotometer (Thermo Fisher Scientific).

Total cellular proteins were determined using the BCA protein assay (Thermo Fisher Scientific) by measuring the absorbance at 595 nm. ALP activity values were normalized to the relative concentrations of total cellular protein.

NO was measured as nitrite

Supplementary References

1. Yamaza, T. *et al.* Mouse mandible contains distinctive mesenchymal stem cells. *J. Dent. Res.* **90**: 317-324 (2011).
2. Yamaza, T. *et al.* Immunomodulatory properties of stem cells from human exfoliated deciduous teeth. *Stem Cell Res. Ther.* **1**: 5 (2010).

Supplementary Table 1. TaqMan probes used in this study.

Gene name	Assay ID
alkaline phosphatase (*Alp*)	Rn01516028_m1
bone gamma-carboxyglutamic acid-containinig protein (*Bglap*)	Rn00566386_g1
bone sialoprotein (*Bsp*)	Rn00561414_m1
collagen, type I, alpha 1 (*Col1a1*)	Rn01463848_m1
dentin matrix protein 1 (*Dmp1*)	Rn01450122_m1
dentin sialophospho protein (*Dspp*)	Rn02132391_s1
nuclear factor kappa B subunit 2 (*Nfkb2*)	Rn01413842_g1
runt-related transcription factor 2 (*Runx2*)	Rn01512298_m1
tumor necrosis factor alpha (*Tnfa*)	Rn01525859_g1

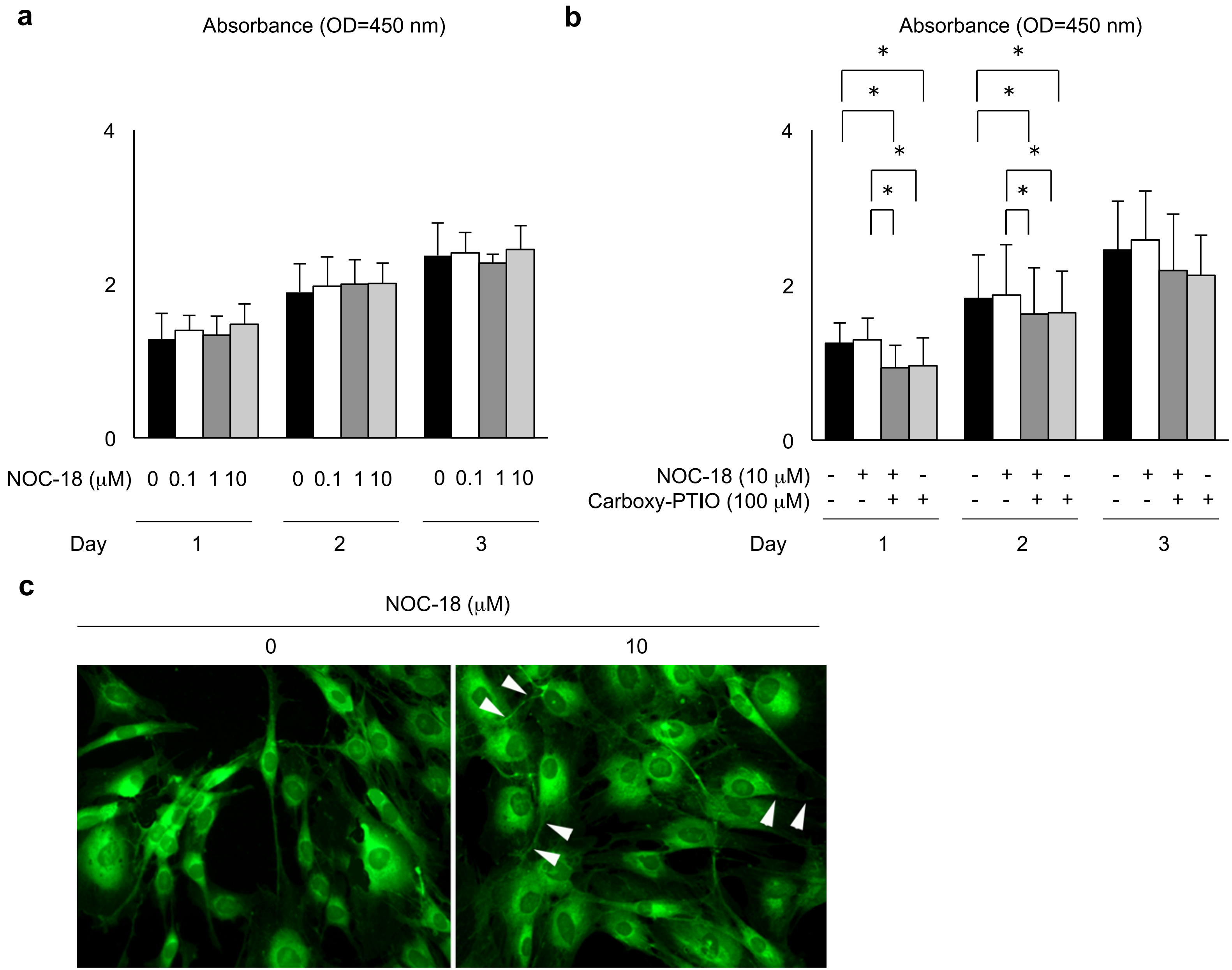

Supplementary Figure 1. Effects of a NO donor, NOC-18, on the cell viability and cellular morphology of rDPSCs. (**a**) Effects of NOC-18 on cell viability of rDPSCs. (**b**) Effects of carboxy-PTIO on cell viability of rDPSCs. (**a, b**) n=3 per group. *: $p<0.05$. Graph bars show the means ± SEM. (**c**) Effects of NOC-18 on cellular morphology of rDPSCs. Cell membrane staining. White arrowheads: a long cytoplasmic process.

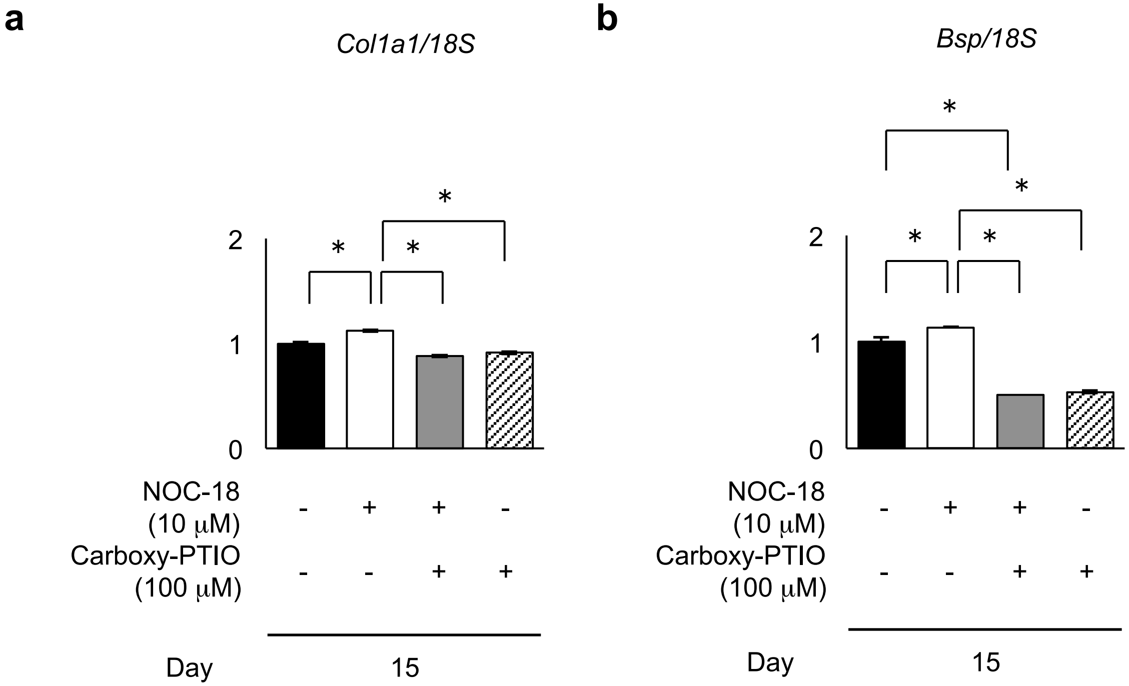

Supplementary Figure 2. Effects of NOC-18 and carboxy-PTIO on gene expression of type I collagen and bone siaoprotein in rDPSCs. rDPSCs were cultured in an odontogenic condition with NOC18 (10 μM) and/or carboxy-PTIO (100 μM). (**a, b**) Real-time RT-PCR assay of type I collagen, type 1, alpha1 (*Col1a1*) (**a**) and bone siaoprotein (*Bsp*) genes (**b**). n=3 per group. Each levels normalized to 18S ribosomal RNA (*18S*) in each sample. Graph bars show the means ± s.e.m. *: $p<0.05$.

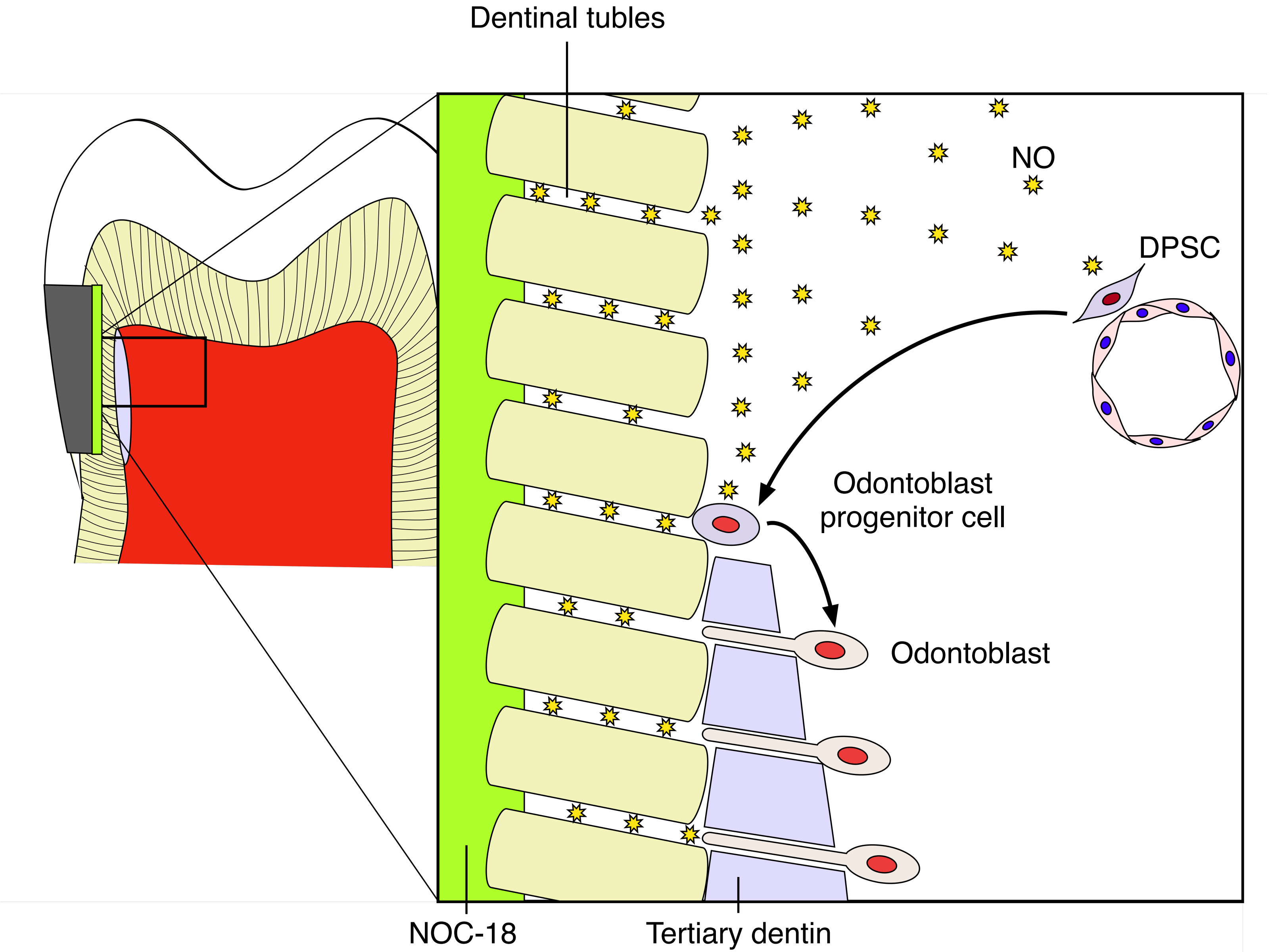

Supplementary Figure 3. Pulp capping application of NOC-18 on the cutting dentin. After tooth preparation, DPSCs are recluited from the perivascular niche to the interface between the injured dentin and dental pulp tissue, resulting in being differentiated into mature odontoblasts to form tertiary dentin. NOC-18-released nitric oxide (NO) spreads in dental pulp tissue beneath the cavity through the dentinal tubles, and accelerates the odontogenic differentiation of the recluited DPSCs.
